# Alpha-fetoprotein (AFP) as tumor marker in a patient with urothelial cancer with exceptional response to anti-PD-1 therapy and an escape lesion mimic

**DOI:** 10.1186/s40425-018-0394-y

**Published:** 2018-09-12

**Authors:** Johannes C. Melms, Rohit Thummalapalli, Kristin Shaw, Huihui Ye, Leo Tsai, Rupal S. Bhatt, Benjamin Izar

**Affiliations:** 1 Division of Hematology and Oncology, Beth Israel Deaconess Medical Center, Boston, MA USA; 2 Department of Medical Oncology, Dana-Farber Cancer Institute, 330 Brookline Avenue, Boston, MA 02215 USA; 3 Department of Pathology, Beth Israel Deaconess Medical Center, Boston, MA USA; 4 Department of Radiology, Beth Israel Deaconess Medical Center, Boston, MA USA; 5 Broad Institute of MIT and Harvard, Cambridge, MA USA; 6 Ludwig Center for Cancer Research at Harvard, Boston, MA USA

## Abstract

The development of a new lesion in a patient with a complete remission to anti-PD-1 therapy is highly concerning for a drug resistant escape lesion. Here, we present a case of a 62-year-old patient with chemotherapy-resistant metastatic urothelial cancer who had a complete remission to pembrolizumab. The patient’s disease burden tracked closely to serum levels of alpha-fetoprotein (AFP) expressed by the tumor and served as an accurate tumor marker. Surveillance imaging revealed a solitary growing pulmonary nodule mimicking an escape lesion in the absence of an increase in AFP levels. Biopsy of this lesion revealed a benign intraparenchymal lymph node with no evidence of metastatic carcinoma. This case indicates that in some patients, biomarkers aberrantly expressed by their tumors, such as AFP in this patient, may be used as a tumor marker for response to anti-PD-1 therapy and emphasizes the importance of confirming potential escape lesions by pathologic examination.

## Background

Immune checkpoint blockade (ICB), including anti-PD-1/PD-L1 and anti-CTLA-4 therapy, revolutionized the treatment landscape of several cancers, including urothelial cancer [1]. While some patients experience deep and durable responses, the majority of patients have either intrinsic or acquired resistance [2]. Progressive disease is most frequently discovered by enlarging or new lesions on cross-sectional imaging, however, there is a broad differential for nodules, particularly in the lung, including infection and inflammation [3]. Determining the etiology of lung nodules is therefore important, as it may affect medical and potentially curative surgical management in the context of oligometastatic disease. Tumor markers are helpful in some cancers and can help guiding therapies, for example, CA125 in ovarian cancer. However, no such tumor markers are available in most cancers. Here, we describe a case of a patient with metastatic, chemotherapy-resistant urothelial carcinoma with a complete response to treatment with anti-PD-1 monotherapy. The patient’s tumor aberrantly expressed alpha-fetoprotein (AFP), which served as a serum tumor marker that tracked very closely with disease burden. Following a complete resection, the patient developed an isolated, slowly growing lung nodule. Although there was no concomitant increase in AFP, the patient underwent resection of the lesion, which revealed a benign intraparenchymal lymph node, but no malignant disease.

## Case presentation

**Fig. 1:**
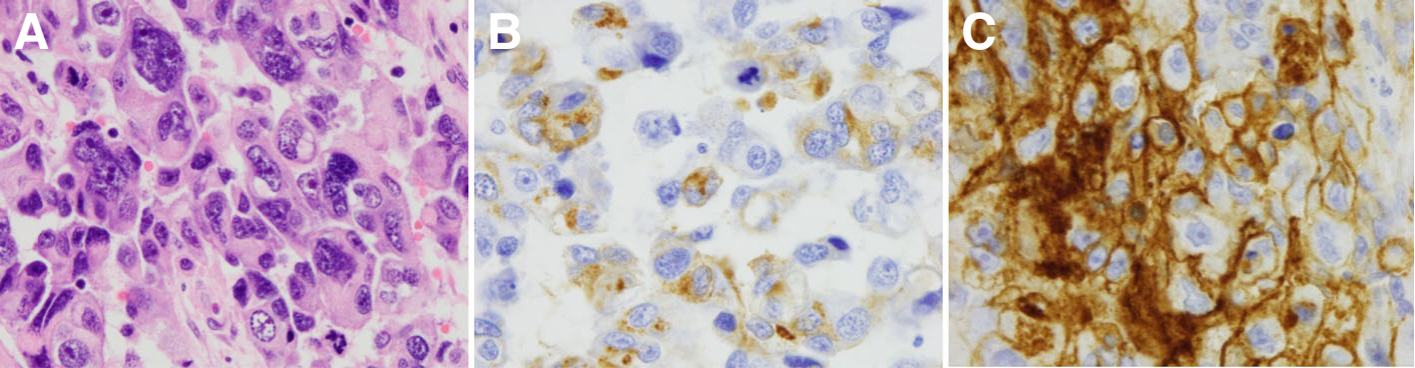
**a** H&E staining of the primary urothelial tumor (40X magnification). **b** Immunohistochemistry (IHC) reveals strong staining of AFP in the primary resection specimen (40X magnification). **c** Staining for PD-L1 shows very strong expression in more than 50% of cancer cells (40X magnification)

**Fig. 2:**
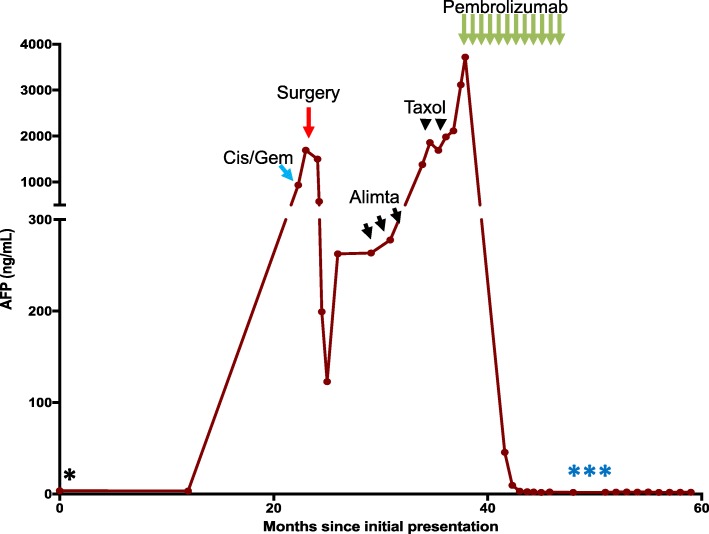
Levels of AFP over the entire clinical course of this patient correlated strongly with tumor burden. The patient had initially (indicated by *) presented with hematuria and concerning cytology, but was lost to follow up. Upon re-presentation with hematuria more than one year later, he underwent full work-up and was found to have urothelial transitional cell carcinoma. Despite receiving neo-adjuvant chemotherapy (cisplatin/gemcitabine), his AFP level strongly increased and he underwent surgery without further delay leading to a sharp decline in AFP levels. Within 3 months following surgery, his AFP level rose again, and after initially declining chemotherapy, he was started on pemetrexed, receiving three cycles total (indicated by short black arrows), however, AFP levels continued to rise. He then received paclitaxel for two cycles (indicated by arrow heads) without response. Ultimately, pembrolizumab was started (indicated by a green arrows) to which he had a sharp decline in AFP levels, significant response on imaging and dramatic clinical improvement. AFP levels normalized after the third infusion. After 12 cycles of pembrolizumab, he was noted to have an isolated lung nodule and underwent wedge resection (indicated by blue ***). He remains off pembrolizumab with continued complete remission and normalized AFP levels

**Fig. 3:**
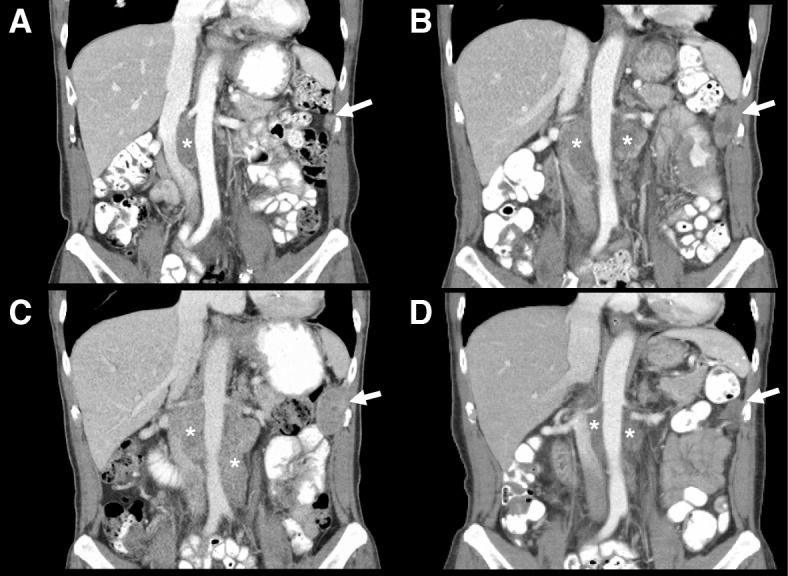
Representative coronal reconstruction from CT scans obtained throughout the clinical course. (*) indicate retroperitoneal lymphadenopathy and white arrows indicate peritoneal metastases. **a** CT scan from initial staging (prior to neo-adjuvant chemotherapy). **b** Progressive disease while on pemetrexed. **c** Further progression on paclitaxel. **d** Significant reduction in tumor burden following third dose of pembrolizumab

**Fig. 4:**
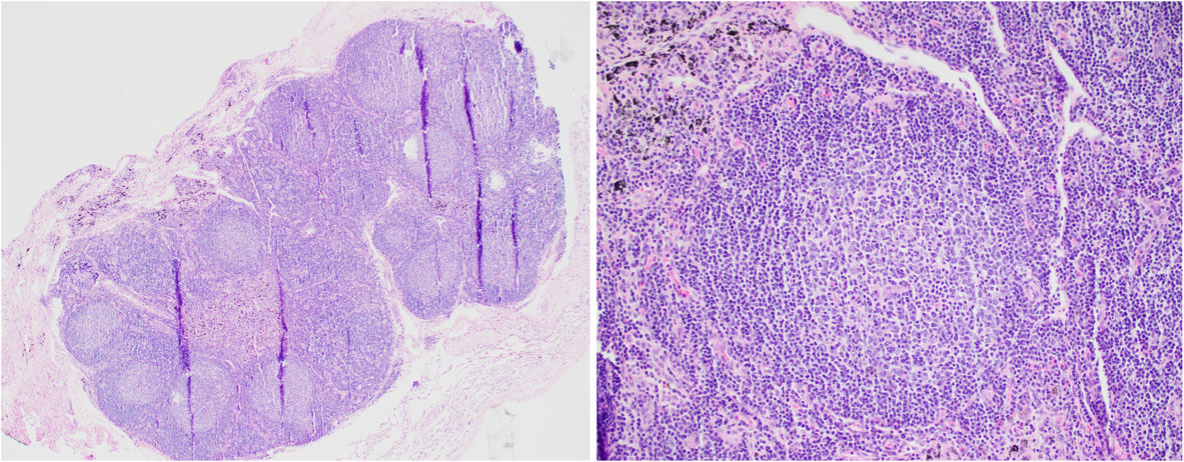
H&E staining of the resected lung lesion, which revealed an intrapulmonary lymph node/lymphoid aggregates (40X magnification, left; 200X magnification, right)

A 62-year-old man with a history significant for chronic hepatitis B initially presented with hematuria and urine cytology positive for malignant cells. A CT urogram revealed a large ill-defined mass of the left posterolateral aspect of the urinary bladder with extramural extension, likely involvement of the seminal vesicles and the prostate, and bilateral enlargement of the external iliac chain lymph nodes. Partial transurethral resection of the tumor was performed, and pathology confirmed an invasive high-grade urothelial carcinoma with squamous differentiation with muscularis propria and lymphovascular invasion. A CT scan of the chest revealed several pulmonary nodules concerning for metastatic disease. An MRI of the liver did not reveal presence of metastatic disease or concern for hepatocellular carcinoma (HCC). Concurrent testing of serum alpha-fetoprotein (AFP) intended for HCC screening (for chronic, but inactive hepatitis B virus infection) showed a very high value of 934.7 ng/mL (normal < 5 ng/mL). He next received neoadjuvant gemcitabine/cisplatin followed by radical cystoprostatectomy and pelvic lymphadenectomy. Pathologic examination revealed a high-grade, poorly differentiated urothelial carcinoma with squamous differentiation with involvement of the left ureter, lymphovascular invasion, extension into perivesical fat, and involvement of 8 of 9 resected lymph nodes. Immunohistochemistry (IHC) of the primary tumor revealed strong staining for AFP (Fig. 1 A-B), confirming tumor-derived AFP production. Serum AFP levels showed a steep decline following surgery, further validating this as a tumor marker, which was subsequently followed throughout the patient’s treatment course (Fig. 2). Molecular testing of the primary tumor using a targeted next-generation sequencing assay (SNaPshot V1) revealed a single nucleotide variant in *TP53* (Arg282Trp). FISH was consistent with amplification of the *HER2* gene, but there were no targetable alterations. On surveillance CT of the abdomen and pelvis three months following surgery, the patient experienced a significant disease relapse, initially deferred initiation of chemotherapy, however, ultimately began treatment with pemetrexed for a total of three cycles (Fig. 2). He continued to demonstrate rising serum AFP levels that correlated with progressive disease, now with palpable metastatic lesions in the head and neck area and continued visceral progression. Finally, he received paclitaxel monotherapy for two cycles, but did not tolerate this therapy well and continued to experience rapidly progressing disease. Histologic examination of his primary tumor revealed strong staining for PD-L1 (Fig. 1C). Due to his chronic hepatitis B infection, however, he was not eligible for clinical trials of immunotherapies. We therefore initiated therapy with the anti-PD-1 checkpoint inhibitor pembrolizumab (2 mg/kg every 3 weeks). Within 6 weeks, his AFP levels dropped from a peak level of ~ 3800 ng/mL to 42 ng/mL. This coincided with a dramatic clinical response, with reduction or resolution of all palpable metastatic lesions. After 4 doses of pembrolizumab, imaging revealed significant shrinkage of all metastatic lesions in the abdomen, including peritoneal masses and mesenteric lymphadenopathy (Fig. 3). Notably, no lung nodules were appreciated on these imaging studies. The patient went on to receive a total of 16 cycles of pembrolizumab and sustained complete remission also reflected by normalized AFP levels, while only experiencing minimal adverse effects. However, after 12 cycles of pembrolizumab therapy, the patient was noted to have a left lower lobe lung nodule measuring ~ 7 mm in largest dimension, which increased to 10 mm on subsequent imaging. Due to concern for a metastatic escape lesion, and in the absence of other evidence of disease, the patient underwent wedge resection of this lung nodule. Of note, this lesion occurred in the absence of AFP elevation. Histopathologic assessment of the resection specimen demonstrated a benign intraparenchymal lymph node with no malignant cells seen (Fig. 4). Since the resection, the patient received no additional systemic therapy and continues to have no evidence of disease for 18 months (as of July 2018).

## Discussion

Immune checkpoint blockade has revolutionized therapy for several different cancer types, however, drug resistance remains a major challenge for the majority of patients [2]. Unfortunately, the majority of patients experience either disease growth or development of new metastatic lesions that escape immunity. It is clinically important to obtain histopathologic confirmation of true progression, as compared to so-called pseudo-progression [4], where lesions grow due to influx of immune cells and increase of biomass on radiographic imaging. Proper identification of new lesions could dictate treatment decisions. In addition to pseudo-progression, one has to distinguish other reasons for new lesions, in particular in the lung, such as infections or inflammation [3].

We report a case of a urothelial cancer patient with complete response to immune checkpoint blockade who developed an apparent escape lesion. The patient’s disease tracked very closely to serum AFP levels, and the strong expression of AFP on the tumor itself supported the tumor as the primary source. AFP is a tumor marker used for screening for hepatocellular carcinoma (HCC) [5] and was the reason that it was checked in this patient with chronic hepatitis B. AFP-producing urothelial tumors are extremely rare [6]. In the case presented here, AFP levels were helpful as a serum tumor marker that correlated tightly with the disease burden observed by clinical examination and cross-sectional imaging. Interestingly, the patient developed what appeared to be a pulmonary escape lesion, but without concomitant elevation in AFP. To determine the nature of the lung lesion, the patient underwent resection and histopathological examination, which revealed the presence of a benign intraparenchymal lymph node that mimicked an escape lesion. This highlights the need for definitive diagnosis of patients with isolated presumed escape lesions to guide proper management, and in this patient, the fact that absent AFP elevation was a good indicator for the non-malignant nature of the lesion. Furthermore, we suspect that in addition to being a mere marker of disease burden, it is also possible that AFP peptides may have served as an epitope linked to the deep response following anti-PD-1 therapy. In line with this hypothesis, a recent report in HCC identified an AFP-peptide (AFP-_158_)-specific T cell receptor (TCR) that promotes dramatic activity in in vitro and in vivo tumor models [7] and the development of AFP-targeted chimeric antigen receptor therapy is underway [8].

## Conclusion

In summary, we present a case of deep response to anti-PD-1 therapy with a tumor that strongly expressed AFP, which served as a reliable tumor marker and possibly as immunogenic antigen. The case also highlights the need for biopsy and careful pathologic examination of apparent escape lesions to guide proper clinical management. 
